# Visual Cross-Modal Re-Organization in Children with Cochlear Implants

**DOI:** 10.1371/journal.pone.0147793

**Published:** 2016-01-25

**Authors:** Julia Campbell, Anu Sharma

**Affiliations:** 1 Brain and Behavior Laboratory, University of Colorado at Boulder, 409 UCB, 2501 Kittredge Loop Road, Boulder, Colorado, 80309, United States of America; 2 Institute of Cognitive Science, University of Colorado at Boulder, 344 UCB, Boulder, Colorado, 80309, United States of America; 3 Department of Speech, Language and Hearing Sciences, University of Colorado at Boulder, 409 UCB, 2501 Kittredge Loop Road, Boulder, Colorado, 80309, United States of America; ARC Centre of Excellence in Cognition and its Disorders (CCD), AUSTRALIA

## Abstract

**Background:**

Visual cross-modal re-organization is a neurophysiological process that occurs in deafness. The intact sensory modality of vision recruits cortical areas from the deprived sensory modality of audition. Such compensatory plasticity is documented in deaf adults and animals, and is related to deficits in speech perception performance in cochlear-implanted adults. However, it is unclear whether visual cross-modal re-organization takes place in cochlear-implanted children and whether it may be a source of variability contributing to speech and language outcomes. Thus, the aim of this study was to determine if visual cross-modal re-organization occurs in cochlear-implanted children, and whether it is related to deficits in speech perception performance.

**Methods:**

Visual evoked potentials (VEPs) were recorded via high-density EEG in 41 normal hearing children and 14 cochlear-implanted children, aged 5–15 years, in response to apparent motion and form change. Comparisons of VEP amplitude and latency, as well as source localization results, were conducted between the groups in order to view evidence of visual cross-modal re-organization. Finally, speech perception in background noise performance was correlated to the visual response in the implanted children.

**Results:**

Distinct VEP morphological patterns were observed in both the normal hearing and cochlear-implanted children. However, the cochlear-implanted children demonstrated larger VEP amplitudes and earlier latency, concurrent with activation of right temporal cortex including auditory regions, suggestive of visual cross-modal re-organization. The VEP N1 latency was negatively related to speech perception in background noise for children with cochlear implants.

**Conclusion:**

Our results are among the first to describe cross modal re-organization of auditory cortex by the visual modality in deaf children fitted with cochlear implants. Our findings suggest that, as a group, children with cochlear implants show evidence of visual cross-modal recruitment, which may be a contributing source of variability in speech perception outcomes with their implant.

## Introduction

Recent advances in cochlear implantation have allowed many children who are born with or acquire severe to profound hearing loss to have accessibility to auditory input and develop speech perception and oral language skills. However, understanding why some implanted children are able to perform at age-appropriate levels while others remain delayed in aspects of speech and language development is critical for clinical intervention, and a focus in research studies [1, 2, 3, 4, 5, 6].

Several factors have been identified that account for significant amounts of variance in speech perception outcomes for cochlear-implanted children. Some of these factors include socioeconomic status, rehabilitative communication strategy, duration of deafness, and age of implantation [2, 7, 8, 9, 10, 11, 12]. For example, we know that age of implantation is a critical factor for harnessing the height of cortical plasticity and for allowing auditory cortical maturation to progress appropriately. The end of the sensitive period for cochlear implantation coincides with the peak of synaptic density in auditory cortex, an indication of maximal plasticity, at approximately 3.5 years of age [13, 14, 15, 16]. Implantation within this time frame results in higher speech perception and language performance outcomes in children [2, 3, 10, 17, 18, 19, 20]. However, despite the identification of key variables important to speech perception and language outcomes, such as sensitive periods for auditory cortical development, a great deal of variance in auditory skill development remains unexplained in children with cochlear implants [3, 21].

Research in profoundly deaf animals and adult humans has described a neurophysiological phenomenon that takes place in auditory deprivation, known as visual cross-modal re-organization [22, 23, 24, 25, 26, 27, 28, 29, 30]. For example, if the cortex is deprived of auditory input during development, specifically before speech and language skills mature, there exists a distinct possibility that the visual cortex will recruit auditory cortical areas for visual processing [22, 24, 25, 26, 31]. However, such compensatory plasticity may pose a challenge for the introduction of audition via a cochlear implant due to the potentially limited resources that may remain for the processing of auditory information [32, 33, 34, 35, 36, 37]. Indeed, adult cochlear implant research literature has described that cross-modal re-organization from the visual modality is linked to deficits in speech perception performance [32, 33, 34, 35, 36, 37], suggesting that this may be another variable influencing speech perception and oral language outcomes in cochlear implanted children.

The purpose of this study was to investigate whether visual cross-modal re-organization is evident in deaf children fitted with cochlear implants and whether this form of compensatory re-organization is related to speech perception outcomes with the implant. We recorded visual evoked potentials via high-density EEG in normal hearing and cochlear-implanted children and performed source localization analyses to observe responsive cortical regions. We then measured speech perception performance in background noise and correlated it with the visual response of the cochlear-implanted children.

## Methods

### Participants

Subjects included 16 cochlear-implanted (CI) children between the ages of 4.95 and 15.43 years. Two children who had additional diagnosis of CHARGE syndrome and Auditory Neuropathy Spectrum Disorder (ANSD) were not included in the study due to compromised neurological processing. Thus, a total of 14 cochlear-implanted children remained in the analysis (see Table 1). Eleven of the 14 cochlear-implanted children had bilateral cochlear implants, and 3 were unilaterally implanted with a hearing aid on the contralateral ear. Two siblings (CI9 and CI10), had a diagnosis of enlarged vestibular aqueduct syndrome (EVAS), associated with progressive hearing loss. The average implant age of the first ear was 3.12 years (standard deviation = +/-2.27 years, range = 0.5–8.42 years), and average age of implantation of the second ear was 6.20 years (standard deviation = +/-3.45 years, range = 1–13.18 years). The EEG and speech perception testing took place at least 1.5 years post-implantation of the first ear to ensure that cortical changes and speech perception were relatively stable. Results from the implanted children were compared to a group of 41 normal hearing children (NH) spanning an approximately similar chronological age range (5.87–14.53 years). All testing was done at the University of Colorado. The University of Colorado Institutional Review Board approved the study, and the parents of all children provided written consent along with the child’s verbal and/or written assent, dependent upon the age of the child. Participants were recruited through advertisements in the community and their parents reported no neurological conditions and normal to corrected vision (i.e., a few children wore glasses).

**Table 1:** Demographic Characteristics of Cochlear-Implanted (CI) Children.

Subject	Age	Age at First CI	Age at Second CI	Duration of First CI Experience	Duration of Second CI Experience
CI1	12.46	2.67	5.42	9.79	7.04
CI2	12.39	1.00	3.33	11.39	9.06
CI3	13.13	0.50	8.09	12.63	5.04
CI4	15.43	1.41	9.26	14.02	6.17
CI5	9.40	1.99	4.36	7.41	5.04
CI6	6.89	2.28	2.90	4.61	3.99
CI7	5.84	4.33	Hearing Aid	1.51	N/A
CI8	11.41	1.61	6.61	9.80	4.8
CI9	13.79	8.42	13.18	5.37	0.61
CI10	11.42	6.14	Hearing Aid	5.28	N/A
CI11	11.58	4.38	8.38	7.20	3.20
CI12	6.44	1.23	2.98	5.21	3.46
CI13	7.42	5.10	Hearing Aid	2.40	N/A
CI14	8.68	2.50	6.38	6.18	2.30

Ages and duration of experience are in years.

### Visual Stimuli

All children were shown a high contrast sinusoidal concentric grating that continually transitioned back and forth into a radially modulated grating or circle-star pattern [33, 38, 39, 40] on a 26-inch flat-screen LCD television at a viewing distance of approximately 42 inches. The circle and star figures were presented 150 times. The star grating was present for 600 ms and was immediately followed by the circle grating, also observable for 600 ms. The morphing of the circle-star pattern provided the percept of apparent motion and shape change to the viewer, theoretically activating the dorsal (‘where’) and ventral (‘what’) visual networks [33, 38, 40, 41]. A total of 300 stimulus presentations were presented, resulting in a recording period of three minutes. The visual evoked potential (VEP) was time-locked to the onset of each star and circle grating. The children were instructed to direct their gaze to the center of the pattern at a black dot during the three minutes. Overall, the children were able to accomplish the task, and any spurious eye artifacts, such as saccades, were removed during EEG analyses.

### EEG Recording and Analyses

All children were fitted with a 128-channel EEG electrode recording net (Electrical Geodesics, Inc.). Children with cochlear implants removed the external processor during testing. Complex visual stimuli were presented via E-Prime® 2.0, stimulus software compatible with Net Station 4 (Electrical Geodesics, Inc). The sampling rate for the EEG recordings was 1 kHz, with an online band-pass filter set at 0.1–200 Hz.

The EEG recordings of each child were high-pass filtered offline at 1 Hz and segmented around each stimulus presentation, with 100 ms pre-stimulus and 495 ms post-stimulus time, then exported from Net Station into EEGLAB [42] using MatLab® (The MathWorks®, Inc., 2010). The data were baseline-corrected to the pre-stimulus period of -100 to 0 ms, and noisy channels were removed from the recording. Artifact rejection set at +/- 100 μV was applied to visual EEG, and data were down-sampled to 250 Hz, altering the post-stimulus time to 492 ms. Data were then re-referenced using common average reference and averaged, and removed channels were replaced with interpolated data via a spherical interpolation algorithm. Finally, the average VEP signal at seven electrodes in the central occipital region [43, 44] were averaged together (70 or O1, 71, 74 and 75 or Oz, 76, 82, and 83 or O2) to create a region of interest (ROI) from which amplitude and latency of VEP peaks for the averaged response were measured. VEP amplitude measurements were recorded as follows: P1 amplitude was measured from P1 peak to N1 peak amplitude, N1 amplitude from N1 peak to P2 peak amplitude, and P2 amplitude from P2 peak to the P2 peak offset amplitude [39, 45, 46, 47]. In the case of a multi-peak VEP, P1 amplitude was measured from the P1 peak to N1a peak amplitude, N1a amplitude from N1a peak to P2a peak amplitude, P2a amplitude from P2a peak to N1b peak amplitude, N1b amplitude from N1b peak to P2b peak amplitude, and P2b amplitude from P2b peak to the P2b peak offset amplitude. Peak latency was defined at the midpoint of the peak. Individual waveforms were averaged together to create a grand-averaged waveform for each group (NH and CI). Group VEP waveforms were low-pass filtered offline at 30 Hz for figure presentation purposes only.

### Current Density Reconstruction

Individual subject concatenated EEG data underwent independent component analysis (ICA) in EEGLAB following artifact rejection and common average referencing [39, 43, 48, 49, 50]. ICA is a statistical procedure that identifies spatially fixed and temporally independent components that underlie the evoked potential [51], and has been successfully utilized to determine cortical sources, including those in cochlear implanted children [39, 43, 49, 50, 52, 51, 53]. Thirty-two independent components underlying the VEP waveform were produced for each subject. Independent components consisting of artifact, such as eye blinks and electrical noise, were rejected. Next, the percent variance of the remaining independent components underlying the VEP peak of interest was calculated in EEGLAB [42]. Only independent components accounting for a majority of the percent variance in the peak of interest were retained and exported into CURRY® Scan 7 Neuroimaging Suite (Compumedics Neuroscan™) for source modeling. In CURRY, the individual participant ICA-pruned data were averaged into groups corresponding to the peak component of interest (e.g. P1 in cochlear-implanted children), and a second ICA was computed on the pruned components from the first ICA analysis to remove additional component artifacts.

Current density reconstructions (CDR) were created for the VEP components using standardized low-resolution brain electromagnetic tomography (sLORETA). sLORETA consists of statistical analyses that include the variance of the cortical generator(s) as well as underlying variance due to recording artifact to produce images (CDR) depicting statistically likely areas within the cortex producing post-synaptic electrical activity [54, 55]. Head models for sLORETA analyses were created using Boundary Element Method (BEM) geometry [56] in CURRY based upon developmental white matter averages in children provided by Wilke et al. [50, 57]. The sLORETA output, or CDR F-Distribution, was represented by a scaled color image. The image was placed on a Montreal Neurological Institute (MNI) child MRI. Sagittal MRI slices were selected to illustrate each CDR.

### Speech Perception in Noise

Speech perception in background noise was measured using the BKB-SIN™ test [58, 59], a clinical assessment of the ability to perceive speech in background noise [60]. Children faced a speaker that was placed at 0° azimuth and repeated two recorded sentence lists (six sentences each) presented at 65 dB Hearing Level (HL). Background noise was increased in 5 dB steps from 25 dB signal-to-noise ratio (SNR) to 0 SNR. The SNR level at which the child could repeat 50% of the sentences was calculated and recorded as threshold. The lower the SNR threshold, the better the performance on the test. Recommended corrections were applied to SNR threshold values to account for differences in processing ability due to age [59]. Cochlear-implanted children wore their implant(s), and if applicable, hearing aid, programmed to their usual settings. SNR threshold values were recorded for 13 of the 14 CI children (one child, CI14, was unable to complete testing) and 27 of the 41 NH children. There was no significant difference in age between the group of CI and NH children who underwent BKB-SIN testing (*t*(38) = 0.234, *p* > 0.05).

## Results

### Visual Evoked Potentials

**Fig 1:**
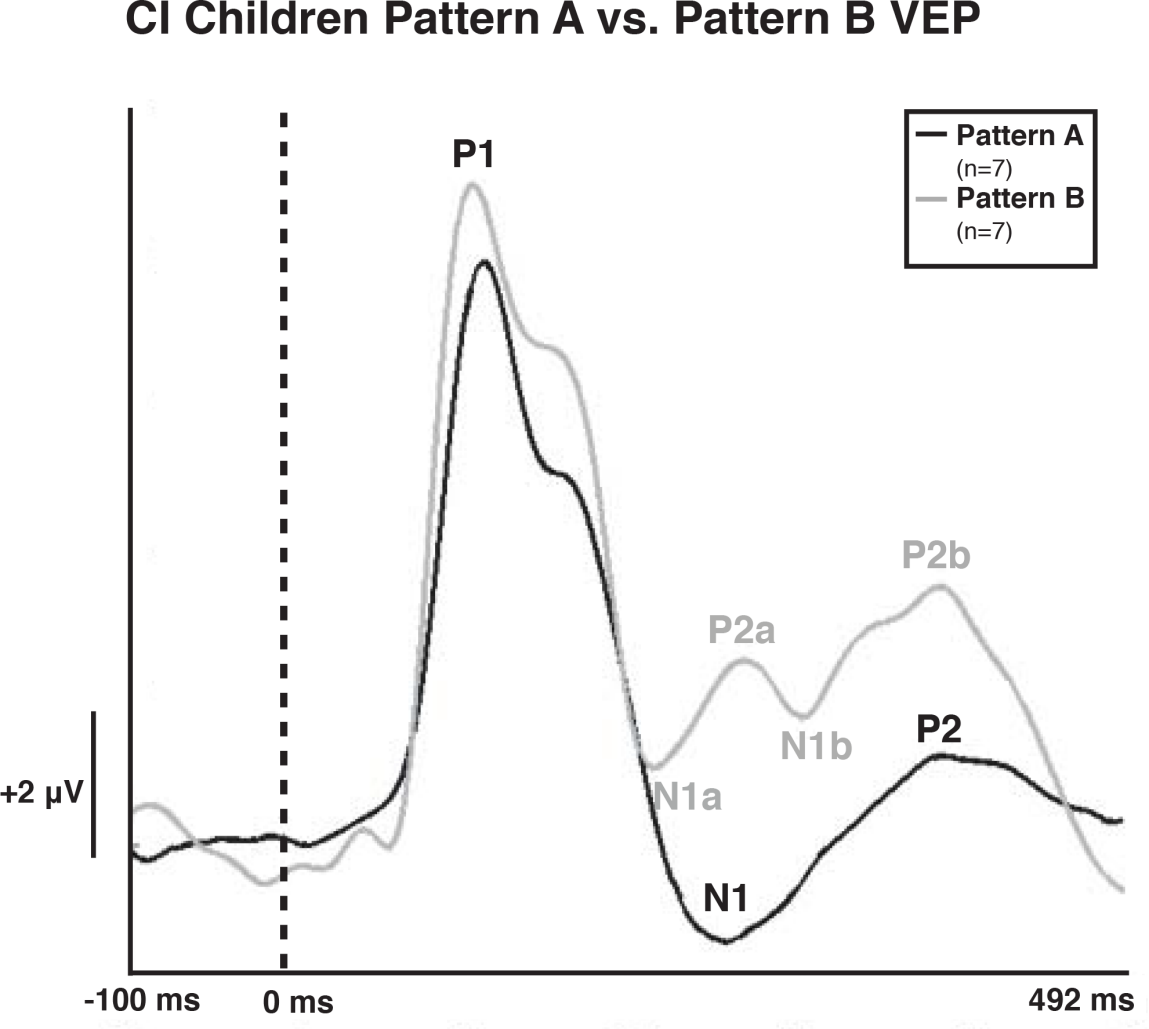
Visual Evoked Potential (VEP) Patterns A and B in 5–15 Year Old Cochlear-implanted (CI) Children. VEP waveforms from the occipital region of interest (ROI) show Pattern A in black and Pattern B in gray. Amplitude is depicted on the vertical axis in microvolts and latency on the horizontal axis in milliseconds. The legend in the upper right shows the number of CI subjects demonstrating VEP pattern A or B according to waveform color.

**Fig 2:**
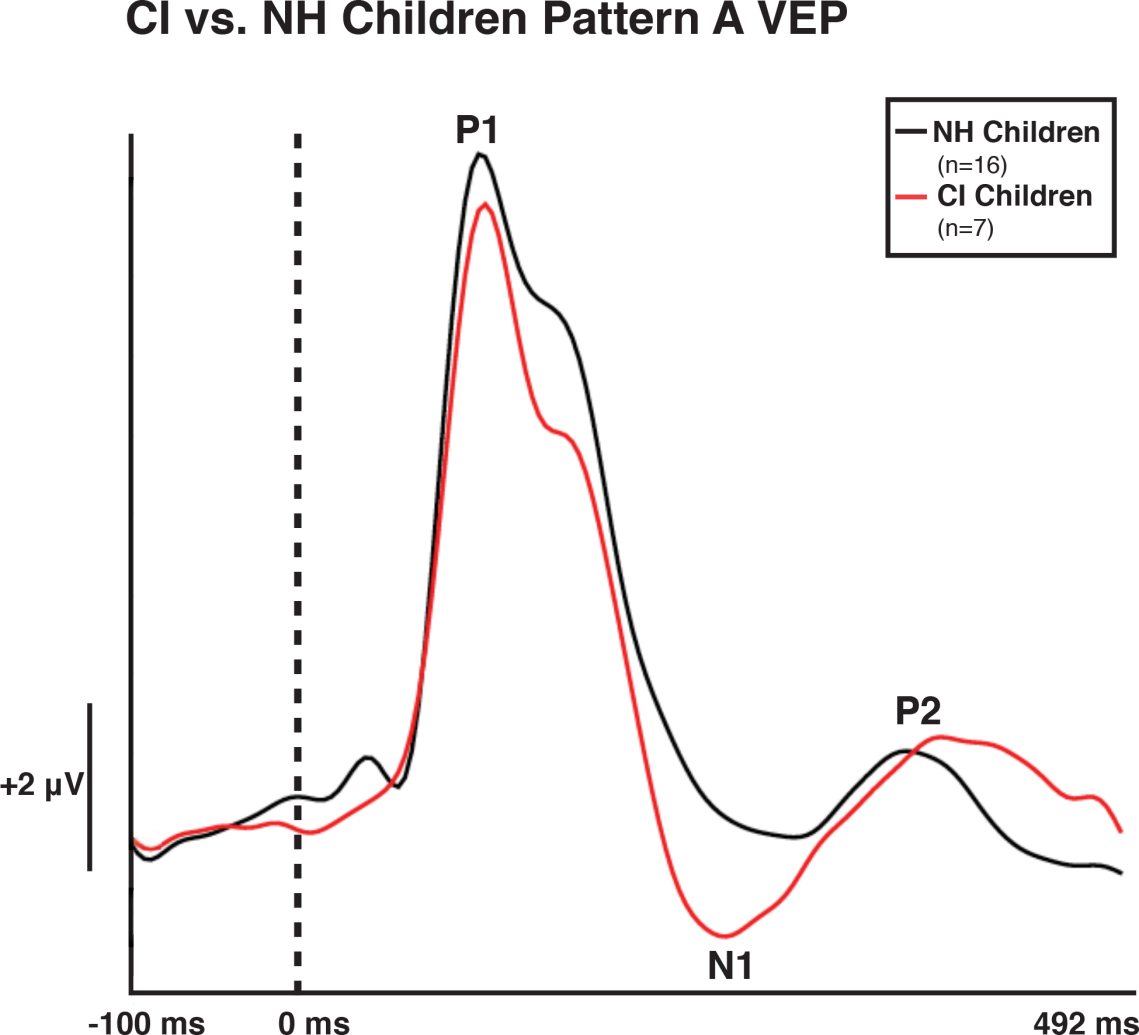
Visual Evoked Potential (VEP) Pattern A in Normal Hearing (NH) and Cochlear-implanted (CI) Children. VEP waveforms from the occipital region of interest (ROI) in 5–15 year old NH children (black) and in 5–15 year old CI children (red). Amplitude is depicted on the vertical axis in microvolts and latency on the horizontal axis in milliseconds. The legend in the upper right shows the number of NH and CI subjects according to respective waveform color.

**Fig 3:**
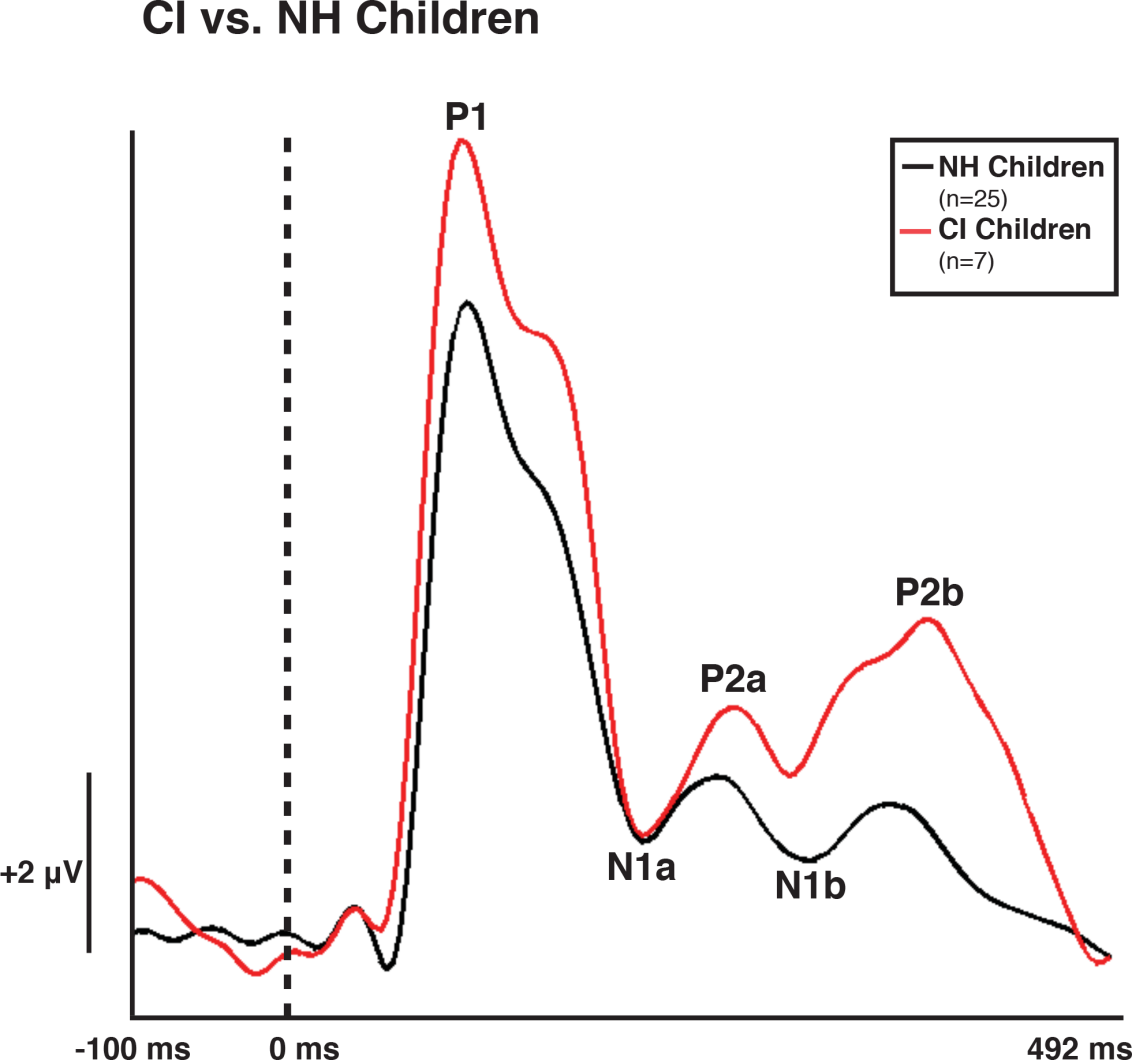
Visual Evoked Potential (VEP) Pattern B in Normal hearing (NH) and Cochlear-implanted (CI) Children. VEP waveforms from the occipital region of interest (ROI) in 5–15 year old NH children (black) and 5–15 year old CI children (red). Amplitude is depicted on the vertical axis in microvolts and latency on the horizontal axis in milliseconds. The legend in the upper right shows the number of NH and CI subjects according to waveform color.

Both NH and CI children demonstrated two morphological patterns of VEP responses (Figs 1–3), which are described below. Given that in previous studies, we have found no developmental differences between the two VEP pattern morphologies in NH children, we created grand average waveforms of each pattern for NH and CI children. Pattern A consisted of three obligatory VEP peak components: P1, N1, and P2. Peak components for pattern A were identified as follows: P1 as the first positive peak occurring at approximately 100 ms, N1 as the first negative peak at approximately 270 ms, and P2 as the second positive peak occurring at approximately 360 ms. The second pattern, pattern B, consisted of multiple peaks labeled as P1, N1a, P2a, N1b, and P2b. Peak components for pattern B were identified as follows: P1 as the first positive peak occurring at approximately 100 ms, N1a as the first negative peak occurring at approximately 200 ms, P2a as the second positive peak occurring at approximately 250 ms, N1b as the second negative peak occurring at approximately 300 ms, and P2b as the third positive peak at approximately 360 ms. VEP morphology similar to that of pattern B (which differs from the typical P1-N1-P2 response) has been identified in the developmental VEP literature, and described in typically developing, non-clinical pediatric populations [40, 61, 62]. Seven CI children (50%) and 16 NH children (39%) presented with pattern A, while 7 CI children (50%) and 25 NH children (61%) presented with pattern B morphology. For each morphological pattern, latencies and amplitudes were compared using the Mann-Whitney U test for one-way ANOVAs [63] and the Kruskall Wallis H test for pairwise comparisons. The Bonferroni correction was used in the case of multiple comparisons.

Fig 1 illustrates the two VEP morphological patterns observed in the CI children. There was no significant difference in age between the children that exhibited pattern A (mean age and standard deviation = 10.79 +/-3.51 years, range = 5.84–15.43 years) and pattern B responses (mean age and standard deviation = 10.11 +/-2.64 years, range = 6.44–13.79 years) (*t*(12) = 0.413, *p* > 0.05). Further, as seen in Fig 1, the P2a peak component in Pattern B is significantly earlier than the P2 peak in Pattern A (*χ*^*2*^(2) = 3.297, *p* = 0.003). This finding indicates that the P2a peak in Pattern B of the CI children possibly occurs as an additional independent component, and that Patterns A and B are distinct from one another, consistent with NH children tested in previous studies in our laboratory.

Fig 2 depicts the comparison between the VEP pattern A response of NH and CI children. There was no significant difference in age between the two groups for this pattern (*t*(21) = -0.719, *p* > 0.05). There was no significant difference between P1, N1 and P2 component latencies or amplitudes for the NH and CI children exhibiting pattern A responses, although the N1 shows an observable trend towards an earlier latency and larger amplitude in the CI children.

Fig 3 shows the comparison between the VEP pattern B response of NH and CI children. There was no significant difference between age for the two groups (*t*(30) = 0.454, *p* > 0.05). While there was no significant difference in P1, N1 and P2 latencies between the two groups, N1a amplitude was significantly greater in the CI group (*U* = 147, *Z* = 2.712, *p* = 0.005), as was P2b amplitude (*U* = 140, *Z* = 2.393, *p* = 0.015). This finding is consistent with studies that have shown larger VEP responses for adults with hearing loss (including cochlear implant users) relative to age-matched normal hearing subjects [32, 33, 39, 64, 65].

### Current Density Reconstruction

Current density reconstruction (CDR) was performed using standardized low-resolution brain electromagnetic tomography (sLORETA) for all VEP components. In order to maximize the number of subjects and statistical power needed to accurately examine visual cortical activation for NH and CI children, similar underlying peak components (as identified through ICA) were combined across VEP patterns and used for CDR analyses. For example, the independent components comprising the P1 peak in the pattern A VEP response were combined with the independent components underlying the P1 peak in the pattern B VEP response for NH and CI children separately. The same procedure was followed for combining the N1 in pattern A and the N1a in pattern B, the N1 in pattern A and N1b in pattern B, and the P2 in pattern A with the P2b in pattern B. As described previously, given that the P2a in pattern B was statistically different from the P2 component in pattern A, the components of these two peaks were not combined. Resulting CDR activations were plotted on an average MRI (sagittal slice view) and the MNI co-ordinates designated beneath each slice. The F-Distributed scale, indicating the strength of the cortical response, is also shown. Cortical regions are listed according to approximate order of response strength in the table to the right of each panel.

**Fig 4:**
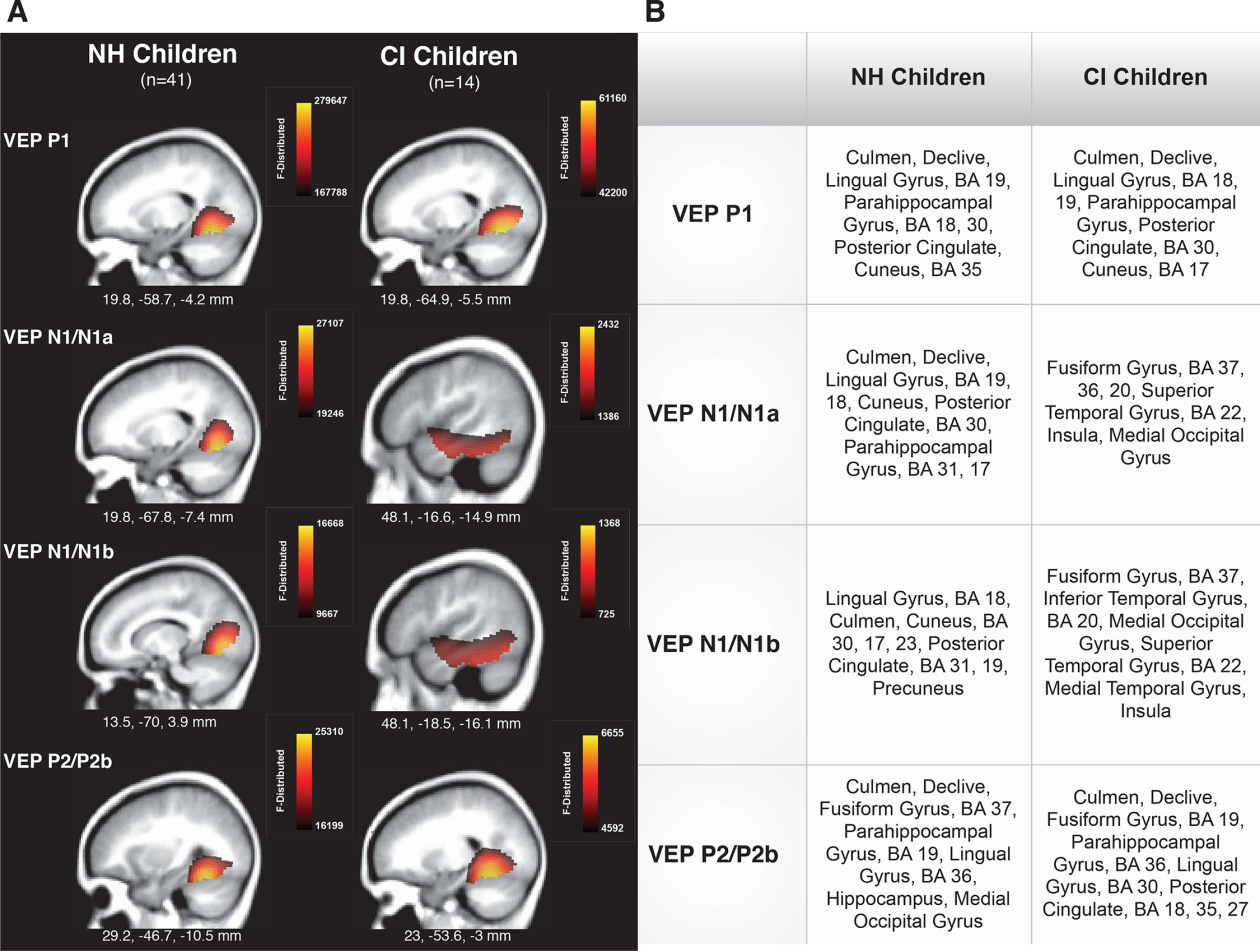
Current Density Reconstructions (CDR) for Visual Evoked Potentials (VEP) in Normal Hearing (NH) and Cochlear-implanted (CI) Children. (A) Cortical activations for each VEP peak component (P1, N1, P2) are shown for NH and CI groups. CDR images are presented on sagittal MRI slices, with three-dimensional Montreal Neurological Institute (MNI) coordinates beneath each slice. The F-Distributed scale is shown for each CDR to the upper right of the MRI. (B) A table listing responsive cortical regions in order of highest to lowest activation for each VEP component in each group.

Fig 4 (Panels A and B), shows the cortical generators in response to visual stimuli for CI children in comparison to NH children. The underlying sources of the VEP P1 peak component are similar for the two groups, with both cerebellar, striate, and extrastriate activations, including cuneus, lingual gyrus, and Brodmann areas 18 and 19. These activations are consistent with other visual imaging studies in adults and children using visual stimuli similar to ours [38, 39, 66, 67]. For the N1/N1a and N1/N1b combined components, it can be observed that the CI children, in addition to normal occipital activation, demonstrated a response in the right lateral temporal cortex. This response was notably absent in the left hemisphere. Right temporal visual cortical activation included superior temporal gyrus, medial temporal gyrus, inferior temporal gyrus, and Brodmann Area 22. These areas were not activated in the NH children, who demonstrated the expected cerebellar and extrastriate responses generating the N1 components. The additional activation of temporal areas in CI children (not seen for NH children) is consistent with studies in profoundly deaf adults and cochlear implanted adults and has been considered to be indicative of visual cross-modal re-organization [24, 25, 26, 31, 32, 33, 35, 37, 39, 68]. In contrast to the N1 components, the P2/P2b components demonstrated expected visual cortical responses for both NH and CI children, showing cerebellar, fusiform, and extrastriate activation. It should be noted that since only seven children showed the P2a response, we have not included those results in Fig 4. However, the visual sources for the P2a were very similar to the N1 components, encompassing the ventral visual stream as well as superior temporal gyrus and Brodmann Area 22. This is expected because the P2a response appears in the same timeframe as the N1 components (see Fig 1) and may involve similar underlying independent components.

### Speech Perception in Noise

Auditory perception of speech in background noise was measured using the BKB-SIN test [58, 59]. This measure provides a signal-to-noise (dB SNR) threshold, or how much greater the signal should be in relation to background noise in order for the child to perceive 50% of the words in a sentence list.

**Fig 5:**
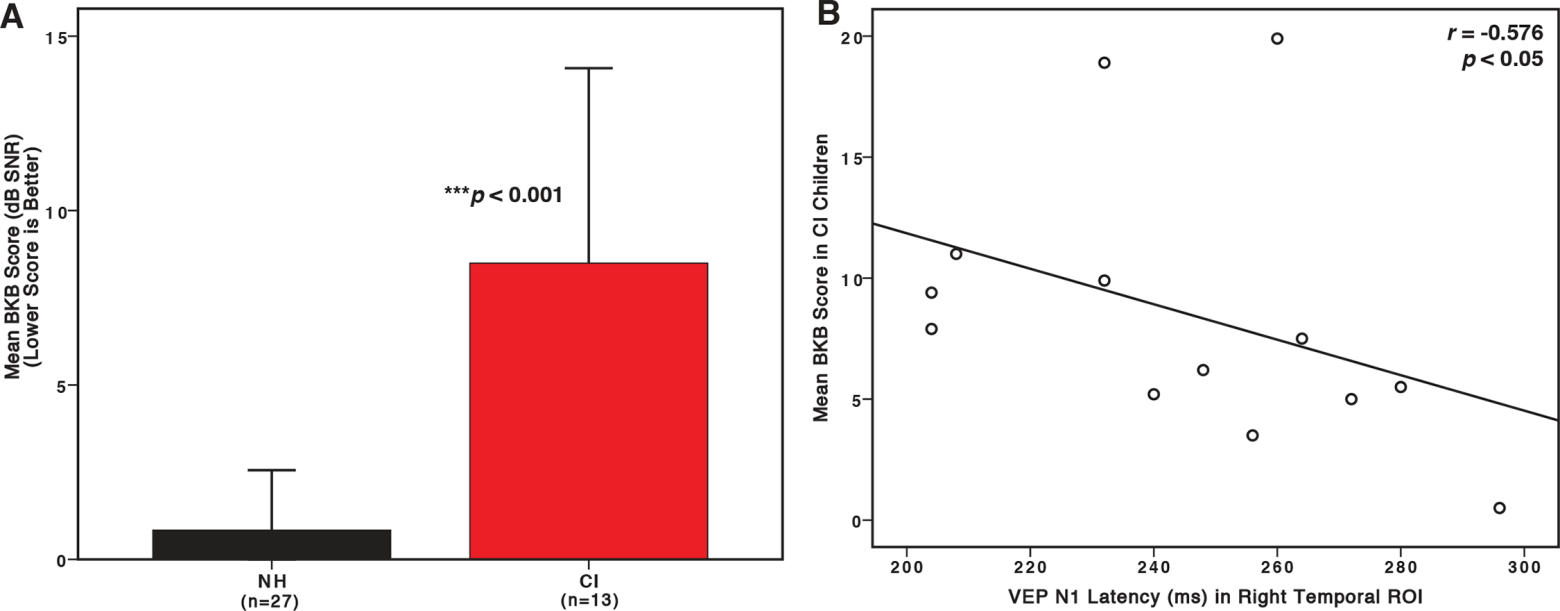
Speech Perception Performance in Background Noise and Visual Cross-modal Re-organization in Cochlear-implanted (CI) children. (A) Mean speech perception in background noise (BKB-SIN) threshold values for NH children and CI children (red) (a lower threshold is better). Error bars depicting one standard deviation are shown as vertical black lines. The asterisk reflects significant difference. (B) Mean BKB-SIN threshold values are shown on the vertical axis and N1 VEP component latencies on the horizontal axis. Values are shown as open circles for the CI group. The Spearman’s rho value and significance are indicated on the upper right hand corner.

As shown in Fig 5A, the CI children had a significantly higher or worse BKB-SIN threshold than the NH children (*U* = 335, *Z* = 4.609, *p* = 0.000). This result is consistent with studies of speech perception in background noise in CI children [69, 70]. In order to correlate the auditory performance in background noise with the visual response in the CI children, the VEP N1 was determined to be an appropriate marker of visual cross-modal plasticity. The VEP N1 response (from the right temporal cortex) has been described as a marker for visual cross-modal plasticity in adults with hearing loss and cochlear implants in previous studies [32, 39], and showed evidence of visual cross-modal re-organization in the CI children in the present study (Fig 4). With this in mind, the average VEP signal at five electrodes in the right temporal region (96 or T6, 97, 101, 102, and 108 or T4) were averaged together to create a region of interest (ROI) in the CI children, and BKB-SIN threshold values were then correlated against the N1 latency of the right temporal ROI.

Fig 5B shows a significant correlation (Spearman’s rank-order) between BKB-SIN threshold and VEP N1 latency (*r* = -0.576, *p* = 0.02). In other words, worse speech perception in background noise was associated with decreased N1 latency. Our finding is congruent with results in cochlear-implanted adults and adults with hearing loss, showing a similar relationship between the VEP N1 component and speech perception performance [32, 39]. Finally, there were no significant differences between the CI children demonstrating VEP pattern A relative to pattern B in regard to mean speech perception thresholds (*U* = 29, *Z* = 1.143, *p* > 0.05).

Due to the finding of significantly larger P2b amplitude in the implanted children concurrent with a localization of visual cortical generators for this component (Fig 4A and 4B), we calculated the correlation (Spearman’s rank-order) between the combined VEP P2/P2b amplitude in the occipital ROI in the CI children with BKB-SIN performance. The resulting correlation approached significance (*r* = 0.473, *p* = 0.051). Thus, worse speech perception in background noise was associated with an increase in VEP P2/P2b amplitude in the occipital region, similar to findings in pre-lingually deaf implanted subjects [71, 72].

## Discussion

In this study, we examined whether cochlear-implanted children would show evidence of cross-modal cortical re-organization from the visual modality and whether such re-organization may be related to speech perception outcomes with the implant. High-density EEG recordings elicited by radially modulated visual gratings and speech perception in noise performance were compared in cochlear-implanted (CI) and normal hearing (NH) children.

We describe three main findings: (i) Both NH and CI children showed two distinct morphological patterns of the VEP response that appear not to be developmentally significant. (ii) The VEP N1 component in the CI children exhibited clear evidence of activation of right temporal cortex, including auditory areas, suggestive of visual cross-modal plasticity. (iii) Decreased VEP N1 latency, considered a marker for cross-modal re-organization, showed a significant inverse relationship with speech perception in noise outcomes for the CI children.

### Converging Evidence for Cross-modal Re-organization From VEP, CDR and Speech Perception

Both the NH and CI children presented with two VEP patterns, illustrating similar morphological development in visual processing of apparent motion and shape change. Pattern A consisted of the typical P1-N1-P2 complex, with a trend toward increased amplitude and decreased latency of the N1 component in the CI children. Pattern B was comprised of a multi-peak response, with the CI children showing larger N1a and P2b amplitude. Larger VEP amplitude, specifically in the N1 component, has been a consistent finding related to visual cross-modal re-organization in profoundly deaf adults, cochlear-implanted adults, and adults with hearing loss [32, 39, 64, 65]. For example, deaf and cochlear-implanted adults have demonstrated increased VEP N1 amplitude over temporal cortex, or an enhanced visual response in this region [32, 64, 65]. Decreased VEP N1 latency has also been linked to visual cross-modal re-organization in adults with hearing loss [39], likely reflecting underlying synaptic changes of visual cross-modal inputs [23, 27].

The CDR results for the N1 component revealed that visual stimuli activated right temporal cortical sources (in addition to normal occipital visual generators) underlying the N1 component in the CI children, but only activated expected or typical visual cortical areas in NH children. Additional areas activated in the CI children included the right ventral visual stream, which is responsible for the processing of object and facial information, as well as auditory processing areas (including superior temporal gyrus and Brodmann Area 22). It is interesting to note that temporal activation was not observed for the left hemisphere. The right temporal hemisphere appears to be more susceptible to visual cross-modal re-organization and its activation by visual stimuli is consistent with such compensatory plasticity reported for adults with hearing loss and cochlear-implanted patients [24, 25, 26, 31, 32, 33, 35, 37, 39]. The finding of activity in the right ventral visual stream and auditory regions is also in agreement with results reported by Giraud and Lee [73], where resting positron emission tomography (PET) data revealed that pre-implantation activation of ventral maladaptive cortical networks were predictive of poor speech perception post-implantation in cochlear implanted children.

In order to investigate whether the observed visual plasticity in the CI children was related to auditory performance, we presented a subset of both groups with sentences in background noise using the BKB-SIN. The CI children performed significantly worse than the NH children in this task (Fig 5A), and their speech perception in noise thresholds reflected a significant negative correlation with N1 latency. The VEP N1 may demonstrate a faster response in temporal cortex, including auditory regions, due to increased synaptic strength and connections involved in visual cross-modal plasticity [23, 27, 74, 75]. Our finding of an inverse relationship between N1 latency and speech perception are consistent with similar findings in hearing-impaired and implanted adults [32, 37, 39, 68] and suggest that visual cross-modal re-organization may negatively impact speech perception performance. Overall, our results indicate that a greater level of visual cortical activity, extending outside of classic visual areas, is related to decreases in speech perception in children with cochlear implants.

In the present study, the latency and CDR of only the VEP N1 component provided clear evidence of cross-modal re-organization by vision. We found no difference in P1 latency, amplitude or CDR sources between the NH and CI children, consistent with similar reports in studies of adults and children with hearing loss and cochlear implants [33, 39]. It is possible that because the P1 reflects primary visual areas, it is less likely to be involved in cross-modal re-organization [33, 40, 76]. Similarly, the CDR for the VEP P2 showed visual sources for both the NH and CI children. However, the P2b showed significantly larger amplitude for the CI children as compared to the NH children. Given that the P2 reflects higher order visual processing, its possible that the larger amplitude in CI children is suggestive of increased visual intra-modal processing. Interestingly, visual intra-modal plasticity, or an enhanced response within the recruiting modality, has also been found to be predictive of poor speech perception in congenitally deaf cochlear-implanted subjects [71, 72, 73]. Along these lines, when we correlated the peak amplitude of the combined VEP P2/P2b components in the CI children from the visual ROI with their auditory performance in background noise, we found a positive correlation approaching significance. That is, increased visual intra-modal plasticity (as reflected by both the increased amplitude and CDR results) was related to poor speech perception in background noise, consistent with previous studies in cochlear-implanted subjects with pre-lingual deafness [71, 72, 73]. This finding, along with visual cross-modal re-organization represented by the N1 components in the CI children, is supportive of an overactive visual system being related to poorer auditory outcomes in congenitally deaf children [71,72]. However, in post-lingual deafness, this visual intra-modal plasticity has been shown to be related to higher speech perception outcomes in implanted subjects, possibly indicating different mechanisms underlying visual plasticity in pre- versus post-lingual deafness [37].

### Visual Cross-modal Recruitment of Right Temporal Cortex

Why is visual cross-modal re-organization of the right temporal cortex implicated in CI children? One reason may be the importance of the right hemisphere ventral visual stream in human facial processing. Subjects who had congenital visual deprivation of the right hemisphere performed more poorly when distinguishing faces than subjects with congenital left hemispheric visual deprivation [77]. In addition, fMRI has shown that the right hemisphere demonstrates a stronger response in both adults and typically developing children during a face-matching task [78]. It is reasonable to assume that in hearing loss, facial processing becomes even more important to satisfy communication needs. Letourneau and Mitchell [79] showed that congenitally deaf adults who use sign language for communication focus equally on bottom and top halves of faces in comparison to normal hearing adults who divided visual attention based on identity or emotion. Behavioral studies in cochlear-implanted children have also revealed the importance of visual facial input during communication. Tyler et al. [80] found that CI children continued to improve in lip-reading several years post-cochlear implantation, while Bergeson et al. [81] reported speech perception performance to be enhanced for CI children when visual cues were also present.

Anatomical changes in the right temporal cortex of subjects with hearing loss may also be related to visual cross-modal re-organization. Structural neuroimaging studies in adults with hearing loss have revealed a loss of gray matter in right auditory cortex [82, 83]. Hearing-impaired adults also show reduced activity in right auditory cortical regions in response to auditory stimuli, concurrent with increased activation of right auditory areas by visual stimuli [39, 43]. Partial or total auditory deprivation may therefore result in a loss or decline of gray matter in auditory regions of the right hemisphere due to lack of stimulation, allowing for greater allocation of resources to visual processing in this area [39, 43].

While evidence of visual cross-modal re-organization appears similar in adults with early-stage, mild-moderate, acquired age-related hearing loss [39], implanted adults with post-lingual deafness [33, 34, 37], and implanted children (as reported in the present study), the underlying mechanisms driving such plasticity are likely different. Cortical plasticity in development is largely driven by extrinsic sensory input, while central auditory and visual pathways have already been created and strengthened in adults with acquired hearing loss [34, 84]. Therefore, visual plasticity as a result of post-lingual hearing loss may be more cognitively-driven (top-down) versus sensory-driven (bottom-up) in pre-lingual deafness [84]. Indeed, cognitive cortical regions are activated by audition in acquired hearing loss, but future studies are needed to ascertain if cognitive cortical regions act as a mediator of visual plasticity [43].

### Synesthesia and Individual Variability

What might be a proposed mechanism by which visual cross-modal recruitment occurs in cochlear-implanted children? Giraud and Lee [73] have proposed that congenital auditory deprivation leads to a synesthetic state of the cortex. If early auditory experience is not present, the auditory cortex is not specifically marked for auditory and speech processing, which may allow inherent cross-modal recruitment by other modalities. The authors suggested that cochlear implantation prior to age 1 year may counteract such effects, though additional research in deaf infants is needed to better understand the prevalence and mechanisms underlying cortical synesthesia. In the present study, the average age of the first implant was 3.1 years, which is relatively late and towards the end of the sensitive period of 3.5 years. It is possible that children implanted at earlier ages, as suggested by Giraud and Lee [73] may not show cross-modal recruitment. Future studies are needed with larger numbers of subjects to examine if there is an effect of age of implantation on cross-modal plasticity. Similarly, future research should also consider other factors such as socioeconomic status, unilateral or bilateral implantation, and rehabilitative communication strategy, among others, which would provide useful information in determining the relative importance of visual cross-modal re-organization to functional outcomes.

In any case, it appears that some CI children may be more prone to cross-modal recruitment. In a recent case report, we describe that cross-modal plasticity by both the visual and somatosensory modality was only evident in children who were average or poor performers with their implant and not in children who were good performers with their implants [85]. This individual variability may have implications for rehabilitation in children with cochlear implants including decision-making on appropriate communication approaches. Overall, our study indicates that visual cross-modal re-organization occurs in CI children and is related to performance with the implant, and should therefore be considered as another factor contributing to the variability in auditory outcomes for cochlear-implanted patients.

## Summary and Conclusion

Our study provides new evidence demonstrative of visual cross-modal plasticity in a group of deaf children fitted with cochlear implants. Converging evidence in the CI children, including decreased latency and increased amplitude of the VEP N1 components, activation of right hemisphere auditory temporal areas underlying the N1 response, and an inverse relationship between VEP N1 latency and speech perception in noise, suggest that, as a group, children with cochlear implants showed evidence of visual cross-modal plasticity. Future studies are needed to determine the full impact of visual cross-modal compensatory plasticity on rehabilitative outcomes for children with cochlear implants, including pre- and post-implantation measures to investigate whether this plasticity is predictive of success with communication approaches and whether it can be enhanced or reversed with training.

## Supporting Information

S1 Table Speech Perception Performance in Background Noise (BKB-SIN) and VEP N1 Latency Data for Fig 5. (A) Subject numbers. (B) Corresponding mean BKB-SIN threshold values. (C) VEP N1 latency (in milliseconds) as recorded from the right temporal region of interest (ROI) for the cochlear-implanted (CI) children.
(XLSX)

## References

[1] Geers AE. Factors influencing spoken language outcomes in children following early cochlear implantation. Adv Otorhinolaryngol. 2006;64: 50–65. 10.1159/000094644 16891836

[2] Niparko JK, Tobey EA, Thal DJ, Eisenberg LS, Wang NY, Quittner AL, et al Spoken language development in children following cochlear implantation. JAMA. 2010;303(15): 1498–1506. 10.1001/jama.2010.451 20407059 PMC3073449

[3] Peterson NR, Pisoni DB, Miyamoto RT. Cochlear implants and spoken language processing abilities: Review and assessment of the literature. Restor Neurol Neurosci. 2010;28(2): 237–250. 10.3233/RNN-2010-0535 20404411 PMC2947146

[4] Sarant JZ, Blamey PJ, Dowell RC, Clark GM, Gibson WP. Variation in speech perception scores among children with cochlear implants. Ear Hear. 2001;22(1): 18–28. 11271973 10.1097/00003446-200102000-00003

[5] Sarant J, Harris D, Bennet L, Bant S. Bilateral versus unilateral cochlear implants in children: a study of spoken language outcomes. Ear Hear. 2014;35(4): 396–409. 10.1097/AUD.0000000000000022 24557003 PMC4072444

[6] Tobey EA, Geers AE, Brenner C, Altuna D, Gabbert G. Factors associated with development of speech production skills in children implanted by age five. Ear Hear. 2003;24(1 Suppl): 36S–45S. 10.1097/01.AUD.0000051688.48224.A6 12612479

[7] Chang DT, Ko AB, Murray GS, Arnold JE, Megerian CA. Lack of financial barriers to pediatric cochlear implantation: impact of socioeconomic status on access and outcomes. Arch Otolaryngol Head Neck Surg. 2010;136(7): 648–657. 10.1001/archoto.2010.90 20644058

[8] Dettman S, Wall E, Constantinescu G, Dowell R. Communication Outcomes for Groups of Children Using Cochlear Implants Enrolled in Auditory-Verbal, Aural-Oral, and Bilingual-Bicultural Early Intervention Programs. Otol Neurotol. 2013;34: 451–459. 10.1097/MAO.0b013e3182839650 23442569

[9] Geers AE. Speech, language, and reading skills after early cochlear implantation. Arch Otolaryngol Head Neck Surg. 2004;130(5): 634–638. 10.1001/archotol.130.5.634 15148189

[10] Kral A, Sharma A. Developmental neuroplasticity after cochlear implantation. Trends Neurosci. 2012;35(2): 111–122. 10.1016/j.tins.2011.09.004 22104561 PMC3561718

[11] Lin FR, Wang N-Y, Fink NE, Quittner AL, Eisenberg LS, Tobey EA, et al Assessing the use of speech and language measures in relation to parental perceptions of development after early cochlear implantation. Otol Neurotol. 2008;29(2): 208–213. 18309575 10.1097/mao.0b013e31812f6fa6 PMC2730755

[12] Wang NY, Eisenberg LS, Johnson KC, Fink NE, Tobey EA, Quittner AL, et al Tracking development of speech recognition: longitudinal data from hierarchical assessments in the Childhood Development after Cochlear Implantation Study. Otol Neurotol. 2008;29(2): 240–245. 10.1097/MAO.0b013e3181627a37 18223451 PMC2733235

[13] Sharma A, Dorman MF, Spahr AJ. A sensitive period for the development of the central auditory system in children with cochlear implants: implications for age of implantation. Ear Hear. 2002a;23(6): 532–539. 10.1097/01.AUD.0000042223.62381.01 12476090

[14] Sharma A, Dorman MF, Spahr AJ. Rapid development of cortical auditory evoked potentials after early cochlear implantation. Neuroreport. 2002b;13(10): 1365–1368. 12151804 10.1097/00001756-200207190-00030

[15] Sharma A, Dorman M, Spahr A, Todd NW. Early cochlear implantation in children allows normal development of central auditory pathways. Ann Otol Rhinol Laryngol Suppl. 2002c;189: 38–41. 12018346 10.1177/00034894021110s508

[16] Huttenlocher PR, Dabholkar AS. Regional differences in synaptogenesis in human cerebral cortex. J Comp Neurol. 1997;387(2): 167–178. 10.1002/(SICI)1096-9861(19971020)387:2<167::AID-CNE1>3.0.CO;2-Z 9336221

[17] De Raeve L. A longitudinal study on auditory perception and speech intelligibility in deaf children implanted younger than 18 months in comparison to those implanted at later ages. Otol Neurotol. 2010;31(8): 1261–1267. 10.1097/MAO.0b013e3181f1cde3 20802371

[18] Harrison RV, Gordon KA, Mount RJ. Is there a critical period for cochlear implantation in congenitally deaf children? Analyses of hearing and speech perception performance after implantation. Dev Psychobiol. 2005;46(3): 252–261. 10.1002/dev.20052 15772969

[19] Holt RF, Svirsky MA. An exploratory look at pediatric cochlear implantation: is earliest always best? Ear Hear. 2008;29(4): 492–511. 10.1097/AUD.0b013e31816c409f 18382374 PMC5494277

[20] Nicholas JG, Geers AE. Will they catch up? The role of age at cochlear implantation in the spoken language development of children with severe to profound hearing loss. J Speech Lang Hear Res. 2007;50(4): 1048–1062. 10.1044/1092-4388(2007/073) 17675604 PMC2882067

[21] Geers AE, Moog JS, Biedenstein J, Brenner C, Hayes H. Spoken language scores of children using cochlear implants compared to hearing age-mates at school entry. J Deaf Stud Deaf Educ. 2009;14(3): 371–385. 10.1093/deafed/enn046 19155289

[22] Bavelier D, Hirshorn EA. I see where you're hearing: how cross-modal plasticity may exploit homologous brain structures. Nat Neurosci. 2010;13(11): 1309–1311. 10.1038/nn1110-1309 20975752

[23] Clemo HR, Lomber SG, Meredith MA. Synaptic Basis for Cross-modal Plasticity: Enhanced Supragranular Dendritic Spine Density in Anterior Ectosylvian Auditory Cortex of the Early Deaf Cat. Cereb Cortex. 2014 10 1 10.1093/cercor/bhu225 PMC4785938 25274986

[24] Fine I, Finney EM, Boynton GM, Dobkins KR. Comparing the effects of auditory deprivation and sign language within the auditory and visual cortex. J Cogn Neurosci. 2005;17(10): 1621–1637. 10.1162/089892905774597173 16269101

[25] Finney EM, Fine I, Dobkins KR. Visual stimuli activate auditory cortex in the deaf. Nat Neurosci. 2001;4(12): 1171–1173. 10.1038/nn763 11704763

[26] Finney EM, Clementz BA, Hickok G, Dobkins KR. Visual stimuli activate auditory cortex in deaf subjects: evidence from MEG. Neuroreport. 2003;14(11): 1425–1427. 12960757 10.1097/00001756-200308060-00004

[27] Kok MA, Chabot N, Lomber SG. Cross-modal reorganization of cortical afferents to dorsal auditory cortex following early- and late-onset deafness. J Comp Neurol. 2013;522(3): 654–675. 10.1002/cne.23439 23897533

[28] Lomber SG, Meredith MA, Kral A. Cross-modal plasticity in specific auditory cortices underlies visual compensations in the deaf. Nat Neurosci. 2010;13(11): 1421–1427. 10.1038/nn.2653 20935644

[29] Meredith MA, Kryklywy J, McMillan AJ, Malhotra S, Lum-Tai R, Lomber SG. Crossmodal reorganization in the early deaf switches sensory, but not behavioral roles of auditory cortex. PNAS. 2011;108(21): 8856–8861. 10.1073/pnas.1018519108 21555555 PMC3102418

[30] Scott GD, Karns CM, Dow MW, Stevens C, Neville H. Enhanced peripheral visual processing in congenitally deaf humans is supported by multiple brain regions, including primary auditory cortex. Front Hum Neurosci. 2014 3 26 10.3389/fnhum.2014.00177/abstract PMC3972453 24723877

[31] Vachon P, Voss P, Lassonde M, Leroux J-M, Mensour B, Beaudoin G, et al Reorganization of the auditory, visual and multimodal areas in early deaf individuals. Neuroscience. 2013;245: 50–60. 10.1016/j.neuroscience.2013.04.004 23590908

[32] Buckley KA, Tobey EA. Cross-modal plasticity and speech perception in pre- and postlingually deaf cochlear implant users. Ear Hear. 2011;32(1): 2–15. 10.1097/AUD.0b013e3181e8534c 20829699

[33] Doucet ME, Bergeron F, Lassonde M, Ferron P, Lepore F. Cross-modal reorganization and speech perception in cochlear implant users. Brain. 2006;129(Pt 12): 3376–3383. 10.1093/brain/awl264 17003067

[34] Lazard DS, Innes-Brown H, Barone P. Adaptation of the communicative brain to post-lingual deafness. Evidence from functional imaging. Hear Res. 2013a 10.1016/j.heares.2013.08.006 23973562

[35] Lazard DS, Lee H-J, Truy E, Giraud A-L. Bilateral reorganization of posterior temporal cortices in post-lingual deafness and its relation to cochlear implant outcome. Hum Brain Mapp. 2013b;34(5): 1208–1219. 10.1002/hbm.21504 22287085 PMC6870107

[36] Sandmann P, Dillier N., Eichele T, Meyer M, Kegel A, Pascual-Marqui RD, et al Visual activation of auditory cortex reflects maladaptive plasticity in cochlear implant users. Brain. 2012;135(2): 555–568. 10.1093/brain/awr329 22232592

[37] Strelnikov K, Rouger J, Demonet JF, Lagleyre S, Fraysse B, Deguine O, et al Visual activity predicts auditory recovery from deafness after adult cochlear implantation. Brain. 2013 10 17 10.1093/brain/awt274 24136826

[38] Bertrand J-A, Lassonde M, Robert M, Nguyen DK, Bertone A, Doucet M-È, et al An intracranial event-related potential study on transformational apparent motion. Does its neural processing differ from real motion? Exp Brain Res. 2012;216(1): 145–153. 10.1007/s00221-011-2920-8 22071683

[39] Campbell J, Sharma A. Cross-modal re-organization in adults with early stage hearing loss. PLoS One. 2014;9: 1–8. 10.1371/journal.pone.0090594.g001 PMC3938766 24587400

[40] Doucet ME, Gosselin F, Lassonde M, Guillemot JP, Lepore F. Development of visual-evoked potentials to radially modulated concentric patterns. Neuroreport. 2005;16(16): 1753–1756. 16237321 10.1097/01.wnr.0000185011.91197.58

[41] Wilkinson F, James TW, Wilson HR, Gati JS, Menon RS, Goodale MA. An fMRI study of the selective activation of human extrastriate form vision areas by radial and concentric gratings. Curr Biol. 2000;10(22): 1455–1458. 11102809 10.1016/s0960-9822(00)00800-9

[42] Delorme A, Makeig S. EEGLAB: an open source toolbox for analysis of single-trial EEG dynamics including independent component analysis. J Neurosci Meth. 2004;134(1): 9–21. 10.1016/j.jneumeth.2003.10.009 15102499

[43] Campbell J, Sharma A. Compensatory changes in cortical resource allocation in adults with hearing loss. Front Sys Neurosci. 2013;7: 1–9. 10.3389/fnsys.2013.00071/abstract PMC3905471 24478637

[44] Stothart G, Tales A, Hedge C, Kazanina N. Double peaked P1 visual evoked potentials in healthy ageing. Clin Neurophysiol. 2014;125(7): 1471–1478. 10.1016/j.clinph.2013.11.029 24370492

[45] Chlubnova J, Kremlacek J, Kubova Z, Kuba M. Visual evoked potentials and event related potentials in congenitally deaf subjects. Physiol Res. 2005;54(6): 577–583. 15717858

[46] Clayson PE, Baldwin SA, Larson MJ. How does noise affect amplitude and latency measurement of event-related potentials (ERPs)? A methodological critique and simulation study. Psychophys. 2013;50(2): 174–186. 10.1111/psyp.12001 23216521

[47] Gilley PM, Sharma A, Dorman M, Martin K. Developmental changes in refractoriness of the cortical auditory evoked potential. Clin Neurophysiol. 2005;116(3): 648–657. 15721079 10.1016/j.clinph.2004.09.009

[48] Debener S, Ullsperger M, Siegel M, Engel AK. Single-trial EEG–fMRI reveals the dynamics of cognitive function. Trends Cog Sci. 2006;10(12): 558–563. 10.1016/j.tics.2006.09.010 17074530

[49] Debener S, Hine J, Bleeck S, & Eyles J. Source localization of auditory evoked potentials after cochlear implantation. Psychophys. 2008; 45(1): 20–24. 10.1111/j.1469-8986.2007.00610.x 17910729

[50] Gilley PM, Sharma A, Dorman MF. Cortical reorganization in children with cochlear implants. Brain Res. 2008;1239: 56–65. 10.1016/j.brainres.2008.08.026 18775684 PMC2783508

[51] Makeig S, Jung TP, Bell AJ, Ghahremani D, Sejnowski TJ. Blind separation of auditory event-related brain responses into independent components. PNAS. 1997;94(20): 10979–10984. 10.1073/pnas.94.20.10979 9380745 PMC23551

[52] Hine J, Debener S. Late auditory evoked potentials asymmetry revisited. Clin Neurophysiol. 2007;118(6): 1274–1285. 10.1016/j.clinph.2007.03.012 17462945

[53] Makeig S, Delorme A, Westerfield M, Jung TP, Townsend J, Courchesne E, Sejnowski TJ. Electroencephalographic brain dynamics following manually responded visual targets. PLoS Biol. 2004;2(6): e176 10.1371/journal.pbio.0020176 15208723 PMC423146

[54] Grech R, Cassar T, Muscat J. Review on solving the inverse problem in EEG source analysis. J NeuroEng and Rehab. 2008;5(25): 1–33. 10.1186/1743-0003-5-25 PMC2605581 18990257

[55] Pascual-Marqui RD. Standardized low-resolution brain electromagnetic tomography (sLORETA): technical details. Methods Find Exp Clin Pharmacol. 2002;24(Suppl. D): 5–12. 12575463

[56] Fuchs M, Kastner J, Wagner M, Hawes S, Ebersole JS. A standardized boundary element method volume conductor model. Clin Neurophysiol. 2002;113(5): 702–712. 11976050 10.1016/s1388-2457(02)00030-5

[57] Wilke M, Krageloh-Mann I, Holland SK. Global and local development of gray and white matter volume in normal children and adolescents. Exp Brain Res. 2007; 178(3):296–307. 10.1007/s00221-006-0732-z 17051378 PMC2265798

[58] Bench J, Kowal Å, Bamford J. The BKB (Bamford-Kowal-Bench) sentence lists for partially-hearing children. British J Audiol. 1979;13(3): 108–112. 10.3109/03005367909078884 486816

[59] Etymotic Research. BKB-SIN™ Speech-in-Noise Test. 2005.

[60] Wilson RH, McArdle RA, Smith SL. An Evaluation of the BKB-SIN, HINT, QuickSIN, and WIN Materials on Listeners With Normal Hearing and Listeners With Hearing Loss. J Speech Lang Hear Res. 2007;50(4): 844–856. 10.1044/1092-4388(2007/059) 17675590

[61] Kubova Z, Kuba M, Kremlacek J, Langrova J, Szanyi J, Vit F, et al Difficulties of motion-onset VEP interpretation in school-age children. Doc Ophthalmol. 2014;128(2): 121–129. 10.1007/s10633-014-9429-y 24563372

[62] Moskowitz A, Sokol S. Developmental changes in the human visual system as reflected by the latency of the pattern reversal VEP. Electroencephalogr Clin Neurophysiol. 1983;56(1): 1–15 6190626 10.1016/0013-4694(83)90002-0

[63] Mann HB, Whitney DR. On a test of whether one of two random variables is stochastically larger than the other. The Annals of Mathematical Statistics. 1947;18(1): 50–60.

[64] Neville HJ, Lawson D. Attention to central and peripheral visual space in a movement detection task: an event-related potential and behavioral study. II. Congenitally deaf adults. Brain Res. 1987;405(2): 268–283. 3567605 10.1016/0006-8993(87)90296-4

[65] Neville HJ, Schmidt A, Kutas M. Altered visual-evoked potentials in congenitally deaf adults. Brain Res. 1983;266(1): 127–132. 6850339 10.1016/0006-8993(83)91314-8

[66] Bucher K, Dietrich T, Marcar VL, Brem S, Halder P, Boujraf S, et al Maturation of luminance- and motion-defined form perception beyond adolescence: A combined ERP and fMRI study. Neuroimage. 2006;31(4): 1625–1636. 10.1016/j.neuroimage.2006.02.032 16624584

[67] Klaver P, Lichtensteiger J, Bucher K, Dietrich T, Loenneker T, Martin E. Dorsal stream development in motion and structure-from-motion perception. Neuroimage. 2008;39(4): 1815–1823. 10.1016/j.neuroimage.2007.11.009 18096410

[68] Chen L-C, Sandmann P, Thorne JD, Bleichner MG, Debener S. Cross-modal functional reorganization of visual and auditory cortex in adult cochlear implant users identified with fNIRS. Neur Plast. 2015;484763:1–13. 10.1155/2016/4382656 PMC4706950 26819766

[69] Caldwell A, Nittrouer S. Speech perception in noise by children with cochlear implants. JSHLR. 2013;56: 13–30. 10.1044/1092-4388(2012/11-0338) PMC3810941 22744138

[70] Gifford RH, Olund AP, Dejong M. Improving speech perception in noise for children with cochlear implants. J Am Acad Audiol. 2011;22(9): 623–632. 10.3766/jaaa.22.9.7 22192607

[71] Lee HJ, Kang E, Oh SH, Kang H, Lee DS, Lee MC, et al Preoperative differences of cerebral metabolism relate to the outcome of cochlear implants in congenitally deaf children. Hear Res. 2005;203: 2–9 15855024 10.1016/j.heares.2004.11.005

[72] Lee HJ, Giraud AL, Kang E, Oh SH, Kang H, Kim CS, et al Cortical activity at rest predicts cochlear implantation outcome. Cereb Cortex 2007;17: 909–17. 16731883 10.1093/cercor/bhl001

[73] Giraud AL, Lee HJ. Predicting cochlear implant outcome from brain organisation in the deaf. Restor Neurol Neurosci. 2007;25(3–4): 381–390. 17943013

[74] Eggermont JJ. On the rate of maturation of sensory evoked potentials. Electroencephalogr Clin Neurophysiol. 1988;70(4): 293–305. 2458238 10.1016/0013-4694(88)90048-x

[75] Eggermont JJ, Ponton CW, Don M, Waring MD, Kwong B. Maturational delays in cortical evoked potentials in cochlear implant users. Acta Otolaryngol. 1997;117(2): 161–163. 9105439 10.3109/00016489709117760

[76] Whittingstall K, Stroink G, Schmidt M. Evaluating the spatial relationship of event-related potential and functional MRI sources in the primary visual cortex. Hum Brain Mapp. 2007;28(2): 134–142. 10.1002/hbm.20265 16761265 PMC6871476

[77] Grand RL, Mondloch CJ, Maurer D, Brent HP. Expert face processing requires visual input to the right hemisphere during infancy. Nat Neurosci. 2003;6(10): 1108–1112. 10.1038/nn1121 12958600

[78] Passarotti AM, Paul BM, Bussiere JR, Buxton RB, Wong EC, Stiles J. The development of face and location processing: an fMRI study. Dev Sci. 2003;6(1): 100–117.

[79] Letourneau SM, Mitchell TV. Gaze patterns during identity and emotion judgments in hearing adults and deaf users of American Sign Language. Perception. 2011;40(5): 563–575. 21882720 10.1068/p6858 PMC3454476

[80] Tyler RS, Fryauf-Bertschy H, Kelsay DM, Gantz BJ, Woodworth GP, Parkinson A. Speech perception by prelingually deaf children using cochlear implants. Otolaryngol Head Neck Surg. 1997;117(3 Pt 1): 180–187. 9334763 10.1016/s0194-5998(97)70172-4

[81] Bergeson TR, Pisoni DB, Davis RA. Development of audiovisual comprehension skills in prelingually deaf children with cochlear implants. Ear Hear. 2005;26(2): 149–164. 15809542 10.1097/00003446-200504000-00004 PMC3432935

[82] Lin FR, Ferrucci L, An Y, Goh JO, Doshi J, Metter EJ, et al Association of hearing impairment with brain volume changes in older adults. Neuroimage. 2014;90: 84–92. 10.1016/j.neuroimage.2013.12.059 24412398 PMC3951583

[83] Peelle JE, Troiani V, Grossman M, Wingfield A. Hearing loss in older adults affects neural systems supporting speech comprehension. J Neurosci. 2011;31(35): 12638–12643. 10.1523/JNEUROSCI.2559-11.2011 21880924 PMC3175595

[84] Kral A., Eggermont J. J. What’s to lose and what’s to learn: Development under auditory deprivation, cochlear implants and limits of cortical plasticity. Brain Res Rev. 2007;56(1): 259–269. 17950463 10.1016/j.brainresrev.2007.07.021

[85] Sharma A, Campbell J, Cardon G. Developmental and cross-modal plasticity in deafness: Evidence from the P1 and N1 event related potentials in cochlear implanted children. Int J Psychophys. 2014;95(2): 135–144. 10.1016/j.ijpsycho.2014.04.007 PMC4209331 24780192

